# Impact of doxorubicin-loaded ferritin nanocages (FerOX) vs. free doxorubicin on T lymphocytes: a translational clinical study on breast cancer patients undergoing neoadjuvant chemotherapy

**DOI:** 10.1186/s12951-024-02441-4

**Published:** 2024-04-15

**Authors:** Marta Sevieri, Francesco Andreata, Francesco Mainini, Lorena Signati, Francesca Piccotti, Marta Truffi, Arianna Bonizzi, Leopoldo Sitia, Claudia Pigliacelli, Carlo Morasso, Barbara Tagliaferri, Fabio Corsi, Serena Mazzucchelli

**Affiliations:** 1 Dipartimento di Scienze Biomediche e Cliniche, Università di Milano, Milan, 20157 Italy; 2 Istituti Clinici Scientifici Maugeri IRCCS, Pavia, 27100 Italy; 3 Division of Immunology, Transplantation, and Infectious Diseases, IRCCS San Raffaele Scientific Institute, Milan, Italy; 4 Laboratory of Supramolecular and Bio-Nanomaterials (SBNLab), Department of Chemistry, Materials, and Chemical Engineering “Giulio Natta”, Politecnico di Milano, Milano, 20131 Italy

**Keywords:** Ferritin nanocages, Doxorubicin, PBMC, T cells, Breast cancer

## Abstract

Despite the advent of numerous targeted therapies in clinical practice, anthracyclines, including doxorubicin (DOX), continue to play a pivotal role in breast cancer (BC) treatment. DOX directly disrupts DNA replication, demonstrating remarkable efficacy against BC cells. However, its non-specificity toward cancer cells leads to significant side effects, limiting its clinical utility. Interestingly, DOX can also enhance the antitumor immune response by promoting immunogenic cell death in BC cells, thereby facilitating the presentation of tumor antigens to the adaptive immune system. However, the generation of an adaptive immune response involves highly proliferative processes, which may be adversely affected by DOX-induced cytotoxicity. Therefore, understanding the impact of DOX on dividing T cells becomes crucial, to deepen our understanding and potentially devise strategies to shield anti-tumor immunity from DOX-induced toxicity. Our investigation focused on studying DOX uptake and its effects on human lymphocytes. We collected lymphocytes from healthy donors and BC patients undergoing neoadjuvant chemotherapy (NAC). Notably, patient-derived peripheral blood mononuclear cells (PBMC) promptly internalized DOX when incubated in vitro or isolated immediately after NAC. These DOX-treated PBMCs exhibited significant proliferative impairment compared to untreated cells or those isolated before treatment initiation. Intriguingly, among diverse lymphocyte sub-populations, CD8 + T cells exhibited the highest uptake of DOX. To address this concern, we explored a novel DOX formulation encapsulated in ferritin nanocages (FerOX). FerOX specifically targets tumors and effectively eradicates BC both in vitro and in vivo. Remarkably, only T cells treated with FerOX exhibited reduced DOX internalization, potentially minimizing cytotoxic effects on adaptive immunity.

Our findings underscore the importance of optimizing DOX delivery to enhance its antitumor efficacy while minimizing adverse effects, highlighting the pivotal role played by FerOX in mitigating DOX-induced toxicity towards T-cells, thereby positioning it as a promising DOX formulation. This study contributes valuable insights to modern cancer therapy and immunomodulation.

**Supplementary Information:**

The online version contains supplementary material available at 10.1186/s12951-024-02441-4.

## Introduction

The anthracycline doxorubicin (DOX) is the first-line therapy for the treatment of different cancers, and is considered a mainstay for breast cancer (BC) treatment [1]. DOX capacity to interfere with DNA replication makes it effective against highly proliferative cells such as cancer cells [2], but it also affects non-malignant dividing cells thus resulting in severe and multifaced off-target toxicities. Besides its direct killing activity and remodulation of the tumor microenvironment [3], DOX induces immunogenic cancer cell death (ICCD) and enhances tumor immunogenicity stimulating the antigen (Ag)-presenting machinery of dendritic cell (DC) [4–7]. DOX-induced ICCD promotes the release of several cancer neoantigens which become accessible to DCs, critical orchestrators of the anti-tumor immune responses [8]. In turn, DCs stimulate rare naïve cancer-specific T cells maturation and clonal expansion, which is necessary to achieve a complete remission and to generate a successful immunological memory that can surveil for relapses and/or metastases [9]. However, at the same time, this antitumor response is restricted by DOX itself. Notably, neutropenia is one of the most prominent hematological toxicities coupled with DOX chemotherapy, and despite it is mostly a transient effect, studies reported how it can cause long-lived immune scars. Since T cell clonal expansion is one of the most proliferative events observed in biological systems, also adaptive immunity is expected to be affected or even suppressed by DOX treatment [10, 11]. This intricate scenario prompts further investigation into the impact of an anthracyclines regimen on dividing T cells to improve DOX immune-mediated antitumor activity [12]. Simultaneously, there is growing interest in developing refined DOX formulations to mitigate detrimental effects such as severe cardiotoxicity resulting from unfavorable pharmacokinetics and inadequate targeting of tumors [13].

In the last 30 years, a plethora of nanoparticle (NP)-based drug delivery systems have been proposed as promising strategies for improving the physicochemical properties of a wide variety of drugs used in oncology, reducing off-site side effects and increasing drug accumulation in target tissues [14–18]. Ferritin nanocages have garnered considerable attention among protein-based NP class due to their innate tumor-homing properties and limited accumulation in non-target organs, which significantly reduces off-target drug toxicity [19]. The use of ferritin nanocages as carriers for encapsulating DOX presents a promising avenue in drug delivery [20]. Ferritin nanocaged DOX leverages the unique structural properties of ferritin, which is a naturally occurring protein that self-assembles into nanoscale cages. These cages provide a controlled and efficient platform for encapsulating DOX. In our study, we introduce FerOX, a DOX formulation within ferritin nanocages. Here, FerOX serves as an alternative nanodrug, preserving the proliferative potential of T cells.

The first aim of this study was to investigate the uptake of DOX and its impact on human lymphocytes considered that, to our best knowledge, its impact on the adaptive immune response remains unclear and insufficiently researched. To this end, we studied DOX uptake and effect on human lymphocytes, firstly derived from healthy donors, then from BC patients that underwent neoadjuvant chemotherapy (NAC). This translational clinical study has shed light on the crucial role of proliferation in generating an effective antitumor response in BC patients. For this reason, alternative formulations of DOX were assessed to gauge their capacity in preserving lymphocyte proliferation. The assessment of DOX-mediated immunotoxicity encompassed evaluations of the FerOX nanoformulation and the clinically available liposomal formulation Myocet. These comprehensive analyses aimed to provide a thorough understanding of how and to what extent T cell clonal expansion, a critical process for generating adaptive immune responses, may be affected following treatment.

## Materials and methods

### Patient recruitment

Thirty patients affected by BC and candidate to NAC with anthracyclines were enrolled in ARMAGEDDON-01 protocol at Istituti Clinici Scientifici Maugeri IRCCS (Pavia, Italy) in the reference period starting from April 2018 to October 2021 (Additional file 1: Table S1; protocol number 2201CE). A written informed consent to participate in the present protocol was obtained from each patient. Venous blood samples were collected in EDTA-coated tubes from each patient before and after (about 3 h) the first cycle of DOX chemotherapy and processed for PBMC isolation within 4 h. As control, a cohort of healthy donors was also enrolled and used in this study. All human samples used in the study were pseudo-anonymized, processed and stored according to standard operating procedures adopted by the institutional biobank “Bruno Boerci” (Istituti Clinici Scientifici Maugeri IRCCS, Pavia, Italy).

### Peripheral blood mononuclear cells collection

Blood samples collected in EDTA-coated tubes were treated by Ficoll gradient to isolate Peripheral Blood Mononuclear Cells (PBMC). Briefly, up to 4.5 mL of blood were gently added to a 15 mL tube containing 5 mL of room temperature (RT) Ficoll (Histopaque®-1077, Sigma) and centrifuged at RT at 400*g* for 30 min in a swinging- bucket rotor without brake. Then, the upper layer of plasma fraction was carefully removed, the mononuclear cell layer containing PBMC was transferred to a 50 mL conical tube and rinsed with 35 mL of phosphate buffer (PBS, Euroclone). PBMC were washed by centrifugation at RT at 400*g* for 10 min. In case of red blood cells contamination, a 5 min treatment in ACK Lysing buffer was performed. PBMC were frozen in Fetal Bovine Serum (FBS, Euroclone) supplemented with 10% DMSO (Sigma) and stored at − 80 °C until usage.

### In vitro* Doxorubicin uptake*

2 × 10^5^ PBMC were seeded in 96 multiwell plates in 200 μL of RPMI medium supplemented with 10% FBS, 1% Penicillin/Streptomycin (Euroclone) and 1% Glutamine (Euroclone) and treated with different concentrations of free Doxorubicin (DOX; 0.01, 0.1, 1 and 10 μM) or with the nanoformulations Myocet and FerOX (1 μM of DOX equivalent). After 1, 3 and 24 h (h), cells were collected and analyzed by flow cytometry (Cytoflex, Beckman Coulter) to quantify DOX mean fluorescence intensity after staining with Live/Dead (L34976; Thermo Scientific). Acquisition was performed on 20,000 events, within the selected region of live singlets. Untreated PBMC were used to select the region of positivity.

To analyze specific cell populations mainly involved in DOX uptake, 2 × 10^5^ PBMC were seeded as reported above and treated with free DOX (1 and 5 μM) or with the nanoformulations Myocet and FerOX (5 μM of DOX equivalent) for 24 h. Then, cells were collected and stained for Live/Dead (L34976, Thermo Scientific), CD3-PECy7 (clone OKT3, Thermo Scientific), CD4-EF506 (clone RPA-T4, Thermo Scientific), CD8a-FITC (clone OKT-8, Thermo Scientific), CD45RA-AF700 (clone HI100, Thermo Scientific) and CD197-APC (clone 3D12, Thermo Scientific). DOX internalization was quantified by flow cytometry. CD4^+^ T-cells were identified as CD3^+^/CD4^+^, while CD8^+^ collected CD3^+^/CD8^+^ cells. T cells subpopulations were identified as follow: Central Memory (CM, CD197^+^ CD45RA^−^), Naïve (CD197^+^ CD45RA^+^), Effector Memory (EM, CD197^−^ CD45RA^−^), Terminally Differentiated Effector (TDE, CD197^−^ CD45RA^+^). Acquisition was performed on 20,000 events, within the selected region of live singlets. Untreated PBMC were used to select the region of DOX positivity. *n* = 3–6.

### Doxorubicin quantification in PBMC from BC patients

PBMC collected from BC patients following the guidelines of Armageddon protocol were analyzed by flow cytometry (Cytoflex, Beckman Coulter) to quantify DOX mean fluorescence intensity after staining with Live/Dead (L34976; Thermo Scientific). To identify subpopulations involved in DOX uptake, cells were stained with CD3-PECy7 (clone OKT3, Thermo Scientific), CD4-EF506 (clone RPA-T4, Thermo Scientific), CD8a-FITC (clone OKT-8, Thermo Scientific), CD45RA-AF700 (clone HI100, Thermo Scientific) and CD197-APC (clone 3D12, Thermo Scientific). T cells subpopulations were identified as follow: Central Memory (CM, CD197^+^ CD45RA^−^), Naïve (CD197^+^ CD45RA^+^), Effector Memory (EM, CD197^−^ CD45RA^−^), Terminally Differentiated Effector (TDE, CD197^−^ CD45RA^+^). Acquisition was performed on 20,000 events, within the selected region of live singlets. Untreated PBMC were used to select the region of positivity.

### Confocal microscopy

1 × 10^5^ PBMC were seeded in 96 multiwell plates in 100 μL of RPMI medium supplemented with 10% FBS, 1% Penicillin/Streptomycin and 1% Glutamine and treated with DOX free (0.1, 1 and 10 μM) or with the nanoformulations Myocet and FerOX (1 μM of DOX equivalent). After 24 h, cells were collected, fixed with 4% paraformaldehyde (PFA, Sigma) for 10 min at RT. Then, cells were labelled with DAPI (1 µM) and mounted on coverslips with ProLong™ Gold (Invitrogen, P36935). Confocal microscopy images were acquired at 1024 × 1024 dpi resolution with the Leica confocal microscope SP8 equipped with 405, 488 and 513 nm lasers.

### CFSE proliferation assay

PBMC from healthy donors or BC patients were marked with CFSE (1 μM in PBS; C34554, Invitrogen, Thermo Fisher) for 10 min at RT and washed twice with RPMI medium supplemented with 10% FBS, 1% Pennicilin/Streptomycin and 1% Glutamine. Cells were then seeded in 96 multiwell round bottom plates (2 × 10^5^ cells in 200 μL of RPMI medium supplemented with 10% FBS, 1% Pennicilin/Streptomycin, 1% Glutamine). PBMC from BC patients collected before and after NAC were analyzed by flow cytometry 2, 3, 4 and 5 days after stimulation with Concanavalin A (ConA, 5 μg/mL, C0412, Sigma). Biological replicates *n* = 6. PBMC from healthy donors were incubated with 5 μM of DOX equivalent (free DOX, Myocet and FerOX) for 3 h and analyzed by flow cytometry at 2, 3, 4 and 5 days after ConA stimulation. Biological replicates *n* = 3. Some PBMC from BC patients collected before chemotherapy were used to simulate an ex vivo treatment and incubated with 5 μM of DOX equivalent (free DOX, Myocet and FerOX) for 3 h and then stimulated with ConA. Three days after ConA treatment, they were labelled with Live/Dead (L34976, Thermo Scientific), CD3-PECy7 (clone OKT3, Thermo Scientific), CD4-EF506 (clone RPA-T4, Thermo Scientific), CD19-SB346 (clone HIB19, Thermo Scientific) and analyzed by flow cytometry. Biological replicates n = 3. The proliferation index was calculated using the software FlowJo (version 10).

### FerOX production

Ferritin nanocages (HFn) were produced in BL21 (DE3) ClearColi® strain and purified by bacterial endotoxins as previously described [21, 22]. FerOX was obtained loading DOX inside HFn using the pH disassembly-reassembly method already described by our group [23]. HFn dissolved at 0.5 mg/mL in 150 mM NaCl were disassembled lowering the pH to 2 and incubating HFn under shaking (180 rpm, RT) for 15 min. Then, 200 μM DOX was added, adjusting the pH back to 7.5 and incubating the mixture for 2 h under shaking (180 rpm, RT). Then, unloaded DOX was removed by centrifugation (3500*g*, 15 min) in 100 kDa Amicon membranes (Millipore) and using 7 K MWCO Zeba™ Spin Desalting columns (Thermo Fisher). The quantification of FerOX content was determined by spectrofluorimetry (FP-800, Jasco) after DOX extraction in isopropanol chloroform solution [24].

Encapsulation efficiency (EE) and loading content (LC) of Dox in HFn were calculated, as widely reported in literature [25], according to the following equations:

$${\text{EE }}\left( \% \right)\, = \,{\text{w1}}/{\text{w2}}\, \times \,{1}00$$

$${\text{LC }}\left( \% \right)\, = \,{\text{w1}}/{\text{w3}}\, \times \,{1}00$$

w1: mass of encapsulated Dox, w2: mass of DOX added to the reaction, w3: mass of HFn used (n = 25). Moreover, according to their relative molecular weights, we calculated the average number of Dox molecules loaded in each HFn nanocage. The EE and loading LC obtained for the FerOX production were 3.33 ± 1.32 and 13.65 ± 5.41, respectively. These correspond to a loading efficiency of 29.38 ± 11.89 molecules of DOX every HFn nanocage, in line with several results reported in literature [26]. Kinetics of DOX spontaneous release in vitro already published in [23] have been confirmed.

### FerOX characterization

The hydrodynamic size of FerOX was studied by Dynamic Light Scattering measurements that were performed on an ALV/CGS-3 Platform-based Goniometer System equipped with an ALV-7004 correlator and an ALV/CGS-3 goniometer. The signal was detected by an ALV-Static and Dynamic Enhancer detection unit. The light source was the second harmonic of a diode-pumped Coherent Innova Nd:YAG laser (λ = 532 nm), linearly polarized in the vertical direction. Measurements were performed at 25 °C. Approximately 1 mL of sample solution was transferred into the cylindrical Hellma scattering cell and data were acquired with a scattering angle set at 90°. Data were analyzed through Laplace inversion (CONTIN algorithm) and both Number averaged distribution and Intensity averaged distribution functions were considered in this study.

The Z-potential was evaluated using a Zetasizer Nano ZS (Malvern Instrument). After equilibrating the sample at 25 °C for 60 s, data were acquired using the following acquisition parameters: three measurements (min 10 max 100 runs/measurement), material (Protein RI 1.450, Absorption 0.001), dispersant (water). Average potential at the slipping plane was obtained using the Smoluchowski correlation.

### Drug screening on patient-derived organoids

Twelve BC Patient-Derived Organoids (PDO), classified as Luminal A (*n* = 4), Luminal B (*n* = 4), HER2^+^-BC (*n* = 2) and Triple-Negative BC (TNBC; *n* = 2), were selected from the “Bruno Boerci” biobank of the Istituti Clinici Scientifici Maugeri IRCCS (Pavia, Italy) and were used to assess FerOX biological activity. PDO, established and cultured as previously described [27], were sheared 2–3 days before seeding to obtain smaller and uniform in size PDO. Briefly, PDO were isolated from Cultrex® Ultimatrix Reduced Growth Factor Basement Membrane Matrix (BME, Bio-techne, BME0010), by incubation with Dispase 1 µg/mL (Gibco, 17,105–041) at 37 °C for 1–2 h. Once the BME was dissolved, PDOs were collected in 15 mL tube and they were washed twice with Ad-DF +  +  + medium (Hyclone DMEM-F/12 1:1 supplemented with 10 mM HEPES, 1% Penicillin/Streptomicin and 1% L-glutamine). The PDO were counted, diluted in culture medium, supplemented with 10% BME and seeded 10.000 cells/well in a 96-wells spheroid microplate (Corning, 4520) at the concentration of 200 cells/µL. After 24 h, 8 different concentrations of DOX, Myocet and FerOX (0.05, 0.1, 0.5, 1, 10, 20, 50, 100 µM) were added in 10 replicates. Untreated cells were used as negative control. After 3 days of incubation at 37 °C and 5% CO_2_, the Cell Titer Glo 3D Kit (Promega, G9682) was used, according to manufacturer’s instructions, to measure the ATP content as an indicator of cell viability. Emitted luminescence was read in microplate reader (PerkinElmer, Victor Nivo Multimode) and data were analyzed using GraphPad Prism 8.

### TfR1 expression on patient derived organoids

3 × 10^6^ organoids were isolated from BME by Dispase 1 µg/mL treatment. Once collected, PDO were reduced into single cells through the shearing procedure using TrypLe™ Select (1 × ; Gibco, 12,563–029). After three washes with HBSS (HyClone Hank’s Balanced Salt Solution), SH30268.02), the cells were fixed with PFA 4% for 5–10 min in ice. Fixed cells were washed thrice with HBSS supplemented with FBS 2% and stained. Staining has been with anti-Transferrin Receptor 1 (TfR1) antibody (1 µg/tube; clone ICO-92; Thermo Fischer Scientific) in PBS, 2% Bovine Serum Albumin (BSA; Sigma) and 2% goat serum (Euroclone) for 30 min at RT. Then, cells were washed thrice with PBS and immunodecorated with Alexa Fluor 488 goat anti-mouse secondary antibody (1 µL/tube; A-11001; Invitrogen; Thermo Fischer Scientific) in PBS, 2% BSA and 2% goat serum for 30 min at RT. After three washes with PBS cells were analyzed by CytoFLEX flow cytometer (Beckman Coulter). 20,000 events were acquired for each analysis, after gating on viable cells and on singlets. The region of positivity has been set using cells immunodecorated only with secondary antibody.

### Statistical analysis

Statistics was evaluated using GraphPad Prism 8.0a version (GraphPad Software Inc., La Jolla, USA). Data are reported as mean ± Standard Error of Mean (SEM). The level of statistical significance was set at p < 0.05. Proliferation index and DOX uptake were analyzed by paired t-test, while One-way ANOVA was used to assess DOX uptake in different T-cells population. Two-way ANOVA was used to study IC_50_ results on PDO considering both the effect of drug and organoid type.

## Results and discussion

### Human peripheral blood mononuclear cells internalized DOX in a dose and time-dependent manner

**Fig. 1:**
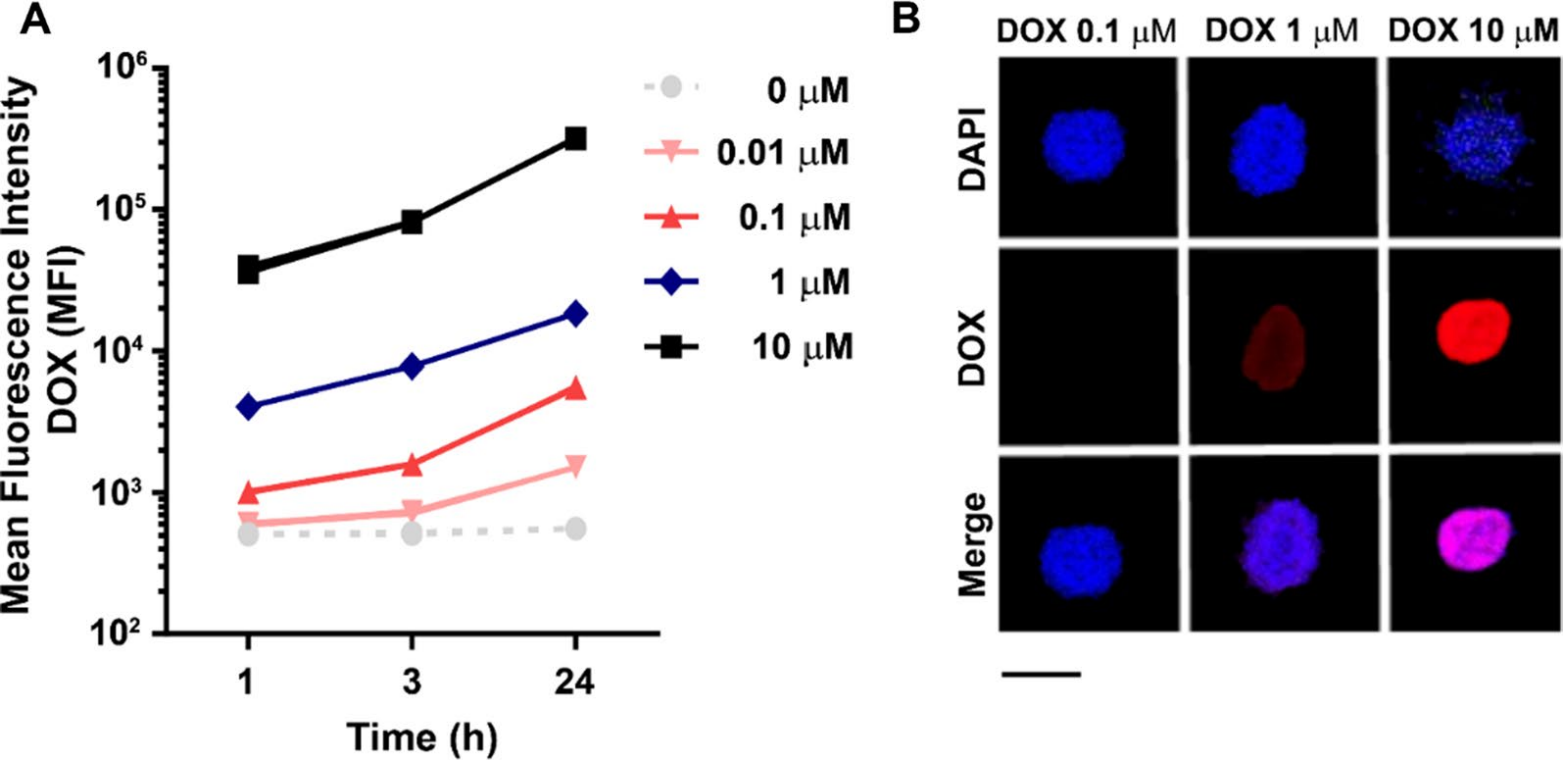
DOX internalization in peripheral blood lymphocytes. **A** PBMC collected from healthy donors and treated with different concentrations of DOX (0.01, 0.1, 1 and 10 μM) for 1, 3 and 24 h to assess drug uptake. DOX uptake has been monitored during time by flow cytometry measuring DOX MFI in DOX^+^ cells. Untreated PBMC were used to select the region of positivity. **B** PBMC treated with DOX at 0.1, 1 and 10 μM for 24 h and acquired by confocal microscopy. Nuclei were stained with DAPI (blue), while the DOX fluorescence signal was reported in red. Scale bar = 10 µm

DOX is an anthracycline chemotherapy drug that works by interfering with the DNA replication process of rapidly dividing cancer cells [3]. DOX is also known to induce ICCD [28], a process that involves the release of various tumor Ag in the tumor microenvironment upon its cytotoxic activity [7]. ICCD requires the involvement of DCs, which capture, process, and display Ag from dying cancer cells to T lymphocytes to unleash an adaptive response [29]. However, the highly-proliferative process of generating an adaptive immune response may be negatively impacted by DOX's cytotoxicity. Therefore, investigating the effect of anthracyclines on dividing T cells is important to determine whether DOX's immunogenicity can be better exploited by developing a nanoformulation that selectively kills tumor cells, while preserving antitumor immune response. In order to do that, we have investigated the effect of DOX on lymphocytes from healthy donors. We found that patient-derived peripheral blood mononuclear cells (PBMC), incubated with DOX (0.01 to 10 μM), displayed a dose and time dependent internalization profile, as evidenced by mean fluorescence intensity (MFI) values of DOX-positive PBMC population analyzed by flow-cytometry (Fig. 1A). Upon internalization, DOX accumulates in the nucleus (Fig. 1B), where it exerts its cytotoxic action by intercalating into the double-helix and disrupting topoisomerase-II-mediated DNA repair [3].

### DOX internalization affects the proliferative potential of human peripheral blood mononuclear cells

**Fig. 2:**
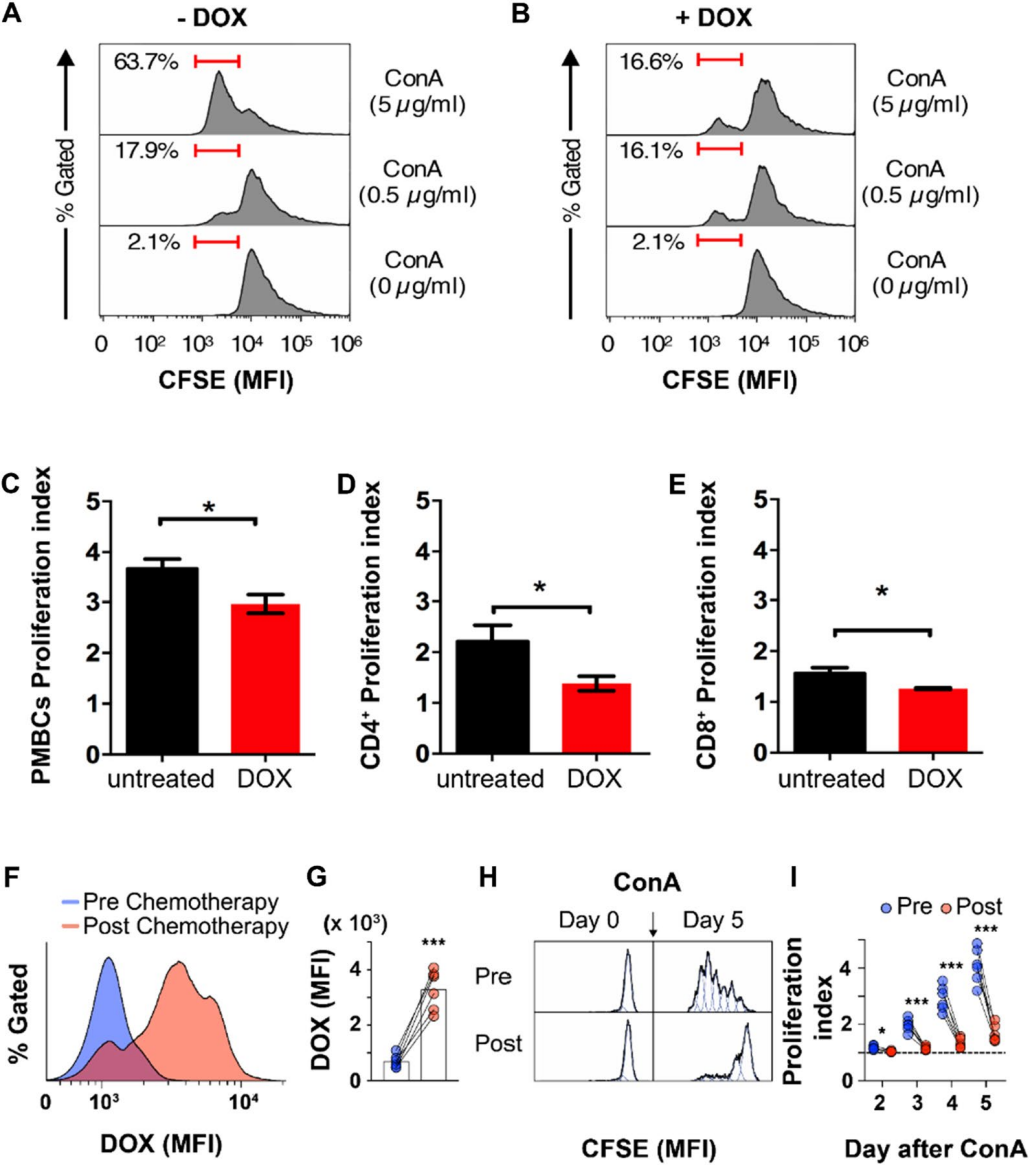
DOX uptake affects peripheral blood lymphocytes proliferation in vitro and ex vivo. **A** Representative proliferation profile of PBMC collected from healthy donors, labelled with CFSE 1 μM and stimulated with Concanavalin A (ConA, 0.5–5 μg/mL). Cells proliferation resulted in reduced CFSE mean fluorescence intensity (MFI). **B** Representative proliferation profile of PBMC collected from healthy donors, labelled with CFSE 1 μM, treated with DOX 5 μM for 24 h and stimulated with ConA (0.5–5 μg/mL). **C** Proliferation index of PBMC after 24 h in vitro treatment with DOX 5 μM calculated using the software FlowJo (version 10). Statistical significance: *p < 0.05 (paired t-test) (n = 3–6). **D** Proliferation index of CD4 + T cells after 24 h in vitro treatment with DOX 5 μM. Statistical significance: *p < 0.05 (paired t-test) (n = 3–6). (**E**) Proliferation index of CD8^+^ T cells after 24 h in vitro treatment with DOX 5 μM. Statistical significance: *p < 0.05 (paired t-test) (n = 3–6). **F** Representative profile of DOX MFI in PBMC collected from BC patient before and after the first cycle of DOX neoadjuvant chemotherapy. (**G**) DOX fluorescence signals detected by flow cytometry in matched PBMC from BC patients collected before and after the first cycle of DOX chemotherapy (n = 6). Statistical significance: ***p < 0.005 (paired t-test). **H** Representative profiles of CFSE MFI signal detected by flow cytometry before and 5 days after ConA stimulation in PBMC population collected from BC patients before and immediately after the first cycle of DOX NAC. **I** Proliferation index calculated at day 2, 3, 4 and 5 after ConA stimulation in matched PBMC collected before and immediately after the first cycle of DOX NAC (n = 6). Statistical significance: *p < 0.05, ***p < 0.005 (paired t-test)

To determine whether the nuclear accumulation of DOX shown in Fig. 1 induces a reduction in proliferation and could thus affect the generation of a successful antitumor immune response, Carboxyfluorescein Succinimidyl Ester (CFSE)-labeled PBMC from healthy donors were exposed to DOX before being treated with the Concanavalin A (ConA) mitogen. In absence of DOX, ConA drove PBMC proliferation in a ConA dose-dependent manner (i.e., 5 and 0.5 μg/mL), as evidenced by the presence of the proliferating PBMC populations with diluted CFSE fluorescence (Fig. 2A). On the other hand, in PBMC previously incubated with DOX, the capability to respond to ConA was strongly reduced (Fig. 2B). Indeed, DOX-treated, ConA-stimulated samples displayed almost a four-fold reduction (i.e., 3.84) in the PBMC population with lower CFSE fluorescence in comparison to that observed in untreated, ConA-stimulated sample (16.6% vs 63.7%; Fig. 2A, B). These results confirmed that DOX uptake is strictly coupled with an impairment of proliferative capability in human PBMCs. After incubating PBMC with 5 μM DOX for 24 h, there was a significant decrease in the proliferation index observed in the overall lymphocyte population (Fig. 2C), as well as in CD4^+^ and CD8^+^ T cells (Fig. 2D, E) showing a global impairment of T-cells mediated immune response after DOX treatment. As PBMC are promptly exposed to DOX during parenteral infusion of DOX-based chemotherapy, we have then evaluated the DOX uptake and the proliferative potential in match-paired PMBC isolated from BC patients before and immediately after (approximatively 3 h after the start of infusion) the first cycle of DOX NAC.

Flow cytometry analysis reported in Fig. 2F–G revealed a DOX^+^ cell population internalizing DOX after chemotherapy and exhibiting a four-fold elevation in DOX MFI. Consistent with results obtained from healthy donors PBMC exposed in vitro to DOX, also BC patient’s in vivo DOX uptake resulted into a significant proliferative impairment in response to mitogenic stimulations, lasting for at least five days (Fig. 2H–I). These results suggest that DOX chemotherapy, while releasing immunogenic tumor Ag upon tumor cells killing, can also affect adaptive antitumor immune response, actually limiting the benefits of ICCD, as already evidenced [30].

To date, it is also crucial to underline that the negative impact of DOX chemotherapy on PBMC derived from BC patients treated with NAC is quick and already detectable after the end of the first cycle of drug infusion. Indeed, performing the count of viable cells collected before and after NAC, it is immediately evident that a marked decrease in total cell number occurs after DOX infusion (Table 1). After infusion, less than half of the circulating immune cells remain viable, yet these surviving cells exhibit a compromised capacity for proliferation (Fig. 2F–I). These results together describe the well-known toxic action of DOX chemotherapy against blood circulating lymphocytes and further indicate that preserving the immune competence is an urgent still unmet clinical need [31, 32].

**Table 1:** Count of viable PBMC collected before and after the first cycle of DOX infusion from 8 BC patients undergoing NAC and recruited in ARMAGEDDON-01 protocol

	PBMC/mL of blood	Ratio
	Mean ± St.dev (× 10^6^)	
Before DOX	1.24 ± 0.67	–
After DOX	0.53 ± 0.15	0.48 ± 0.15

### DOX treatment predominantly targets CD8^+^ T cells

**Fig. 3:**
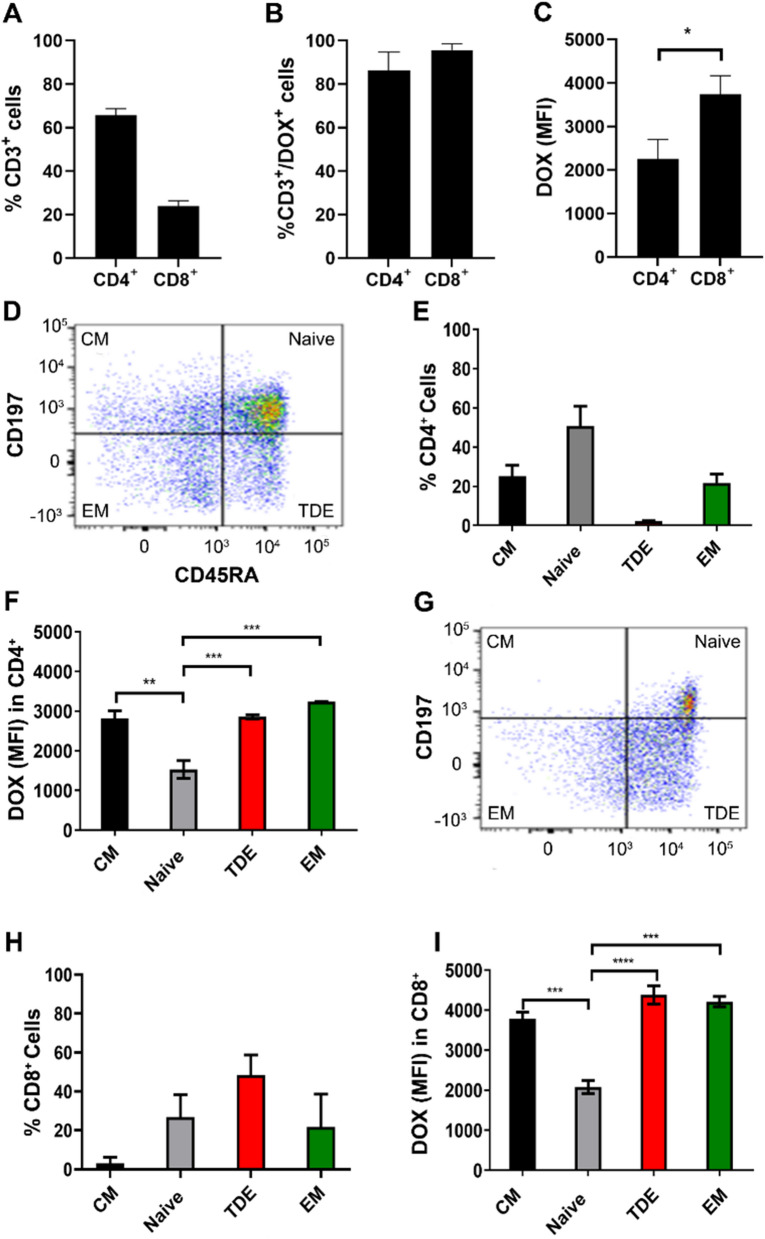
DOX uptake in peripheral blood lymphocytes subpopulations collected from BC patients immediately after the first cycle of NAC. **A** CD8^+^ and CD4^+^ distribution in BC patients’ PBMC collected immediately after the first cycle of NAC. **B** CD8^+^ and CD4^+^ distribution in DOX^+^ population of BC patients’ PBMC collected immediately after the first cycle of NAC. **C** Mean DOX fluorescence intensity in DOX^+^ T cells populations from BC patients’ PBMC collected immediately after the first cycle of NAC. Statistical significance: *p < 0.05 (One-way ANOVA). **D** Representative dot-plot of distribution of CD4^+^ T cells subpopulations from BC patients’ PBMC collected immediately after the first cycle of NAC. **E** CD4^+^ T cells distribution in BC patients’ PBMC collected immediately after the first cycle of NAC. **F** Mean DOX fluorescence intensity in DOX^+^/CD4^+^ T cells subpopulations from BC patients’ PBMC collected immediately after the first cycle of NAC. Statistical significance: **p < 0.01, ***p < 0.005 (One-way ANOVA) (**G**) Representative dot-plot of distribution of CD8^+^ T cells subpopulations from BC patients’ PBMC collected immediately after the first cycle of NAC. **H** CD8^+^ T cells distribution in BC patients’ PBMC collected immediately after the first cycle of NAC. **I** Mean DOX fluorescence intensity in DOX^+^/CD8^+^ T-cells subpopulations from BC patients’ PBMC collected immediately after the first cycle of NAC. Statistical significance: ***p < 0.005, ****p < 0.001 (One-way ANOVA). All assessments have been performed at least on PBMCs collected from at least 3 patients

Since PBMC include also T cells with immunosuppressive activity [33], we have investigated wheter DOX has a preferential effect on a specific T cell population during chemotherapy by analyzing DOX uptake in subsets of CD4^+^ and CD8^+^ cells. Despite the CD3^+^/CD4^+^ population is the more represented in PBMC collected from BC patients (Fig. 3A), CD3^+^/CD4^+^ and CD3^+^/CD8^+^ are almost equally represented in the DOX^+^ population (Fig. 3B). In the meantime, the higher DOX uptake is observed in CD8^+^ population (Fig. 3C), that displayed almost a two-fold DOX MFI, in comparison to CD4^+^ cells, evidencing that CD8^+^ is more prone to DOX internalization than CD4^+^. We then delve into the impact of DOX on T cell subsets, each representing distinct stages of T cell differentiation and activation. This investigation aimed to understand how each subset responds to DOX, providing valuable insights into its potential impact on the adaptive immune response. Specifically, Naïve C ells are undifferentiated T cells crucial for initiating immune responses, while Central Memory T cells already encountered cognate Ag retain the ability to migrate to secondary lymphoid organs and provide rapid response upon re-exposure to the Ag. In contrast, Effector Memory T cells primarily reside in peripheral tissues, also ready for immediate effector functions. Finally, Terminal Differentiated Effector T cells are end-stage effectors with reduced proliferative capacity, playing a significant role in the later stages of the immune response. Among DOX^+^CD4^+^ cells, we observed high frequency of naïve cells (CD45RA^+^CD197^+^), followed by Central Memory (CM, CD45RA^−^CD197^+^) and Effector Memory (EM, CD45RA^−^CD197^−^) cells, while only a little fraction them are Terminal Differentiated Effector (TDE, CD45RA^+^CD197^−^), as reported in Fig. 3D, E. Despite the high percentage of CD4^+^ naïve cells observed in DOX^+^ population, they displayed the lowest DOX MFI, suggesting a lower drug uptake (Fig. 3F). On the contrary, CD4^+^ CM, EM and TDE populations, showed two-fold higher DOX MFI, evidencing that a higher amount of DOX has been internalized in these cells. DOX^+^ cells in CD8^+^ population are mainly subclustered as TDE, followed by Naïve and EM cells, while only a little fraction them are CM (Fig. 3G, H). Also in this case, despite the relatively high percentage of CD8^+^ naïve cells observed in DOX^+^ population, they displayed the lowest DOX MFI, suggesting a reduced drug uptake (Fig. 3I). Similarly, to those observed in CD4^+^ population, CD8^+^ CM, EM and TDE populations, showed two-fold higher DOX MFI, evidencing once again that a higher amount of DOX has been internalized in these cells. These results are consistent with that already observed in the literature, where DOX chemotherapy seems to have a higher negative impact on CD8^+^ T cells [34, 35]. This has been evaluated in T-cells isolated from mouse splenocytes, where CD4^+^ T cells were found to preserve higher proliferation activity after pre-incubation with DOX [34, 35]. Contrary to what encounters for CD8^+^ T cells, DOX may have an impact in the activation of Ag -specific CD4^+^ T cells resulting in a more robust proliferation [34].

### DOX formulation in ferritin nanocages (FerOX) preserves DOX activity in a panel of patient-derived organoids

**Fig. 4:**
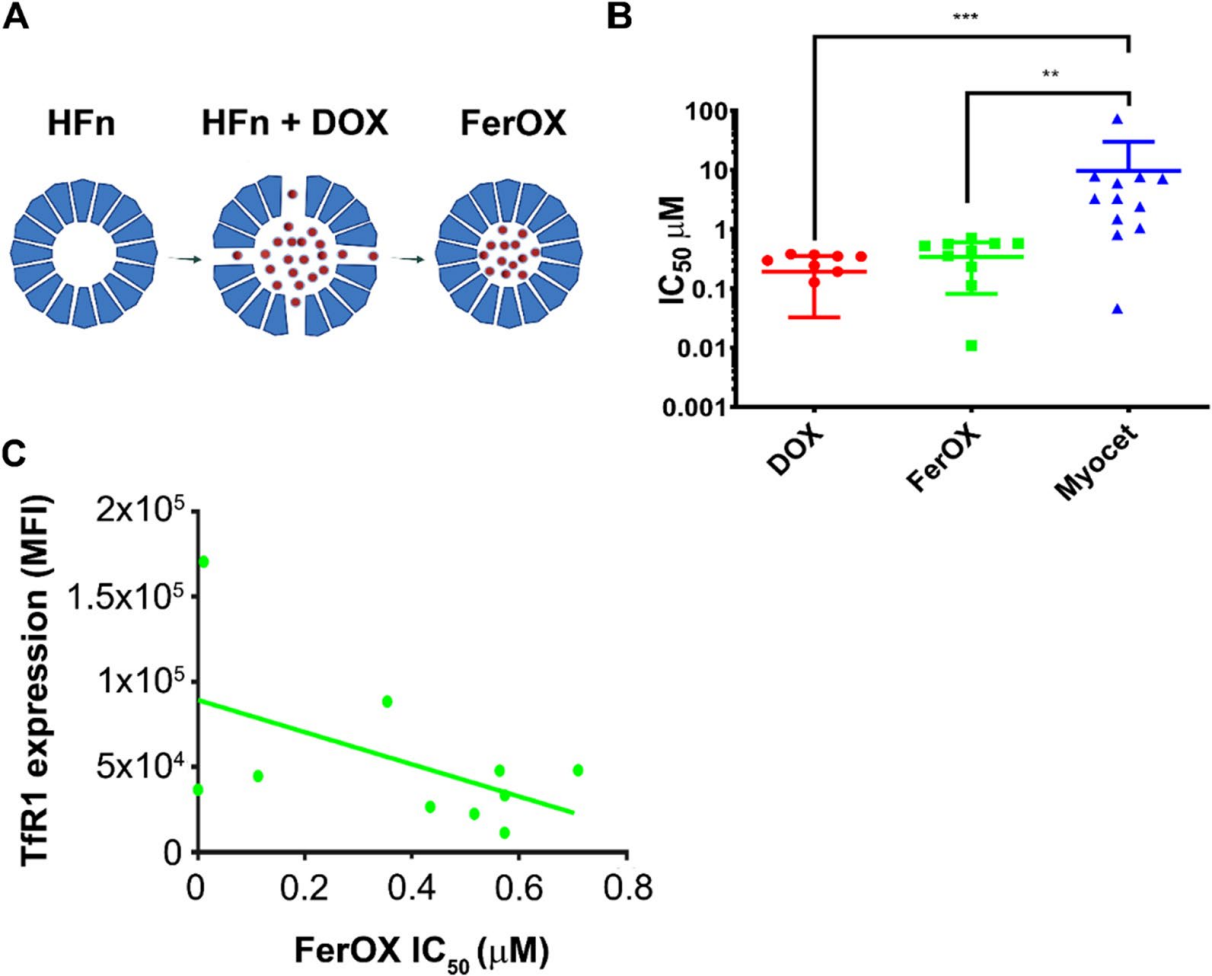
FerOX production and antitumor activity compared to DOX and Myocet. **A** Schematic representation of FerOX production. HFn nanocages have been loaded with DOX following the pH-dependent disassembly/reassembly procedure. **B** IC_50_ of PDO treated with FerOX, DOX and Myocet. PDO’s viability data used to calculate IC_50_ have been obtained by Cell titer Glo assay. Statistical significance: **p < 0.01, ***p < 0.005 (Two-way ANOVA). **C** Correlation between FerOX IC_50_ and TfR1 expression of each PDO (r^2^ = 0.3701; r = − 0.6083; p = 0.0310). TfR1 expression has been determined as mean MFI evaluated by flow cytometry

Results displayed so far provide evidence that DOX uptake varies in CD8^+^ and CD4^+^ subsets in patients, and that this differential uptake impacts the proliferative potential of T cells. As the preservation of proliferative competence is critical for T cell-mediated adaptive antitumor immune response, we opted to evaluate the effect of alternative DOX formulations on these cells. Therefore, we decided to compare a liposomal DOX formulation already introduced in clinical practice (i.e., Myocet), and DOX nanoformulation (i.e., FerOX) [20, 23, 24, 36–38]. FerOX has been extensively studied by nanotechnologists in the past two decades but has not yet been introduced into clinical practice [19, 39]. FerOX is a protein-based nanoformulation of DOX, which exploits the human protein H-ferritin (HFn). Here, DOX is encapsulated within a 12 nm diameter cave sphere constituted by 24 HFn subunits [23]. This nanoplatform is able to specifically recognize the TfR1, which is highly expressed in cancer tissues and mediates the internalization of nanoparticles, thus facilitating the tumor-targeted recognition of this nanoformulation [40]. Thanks to its unique biotechnological properties, HFn quaternary structure could be disassembled lowering the pH until 2.0, and then refolded bringing back the pH to the neutrality [19, 23]. When DOX is added to the solution during this process, the HFn shell is capable of encapsulating it, leading to the production of FerOX (Fig. 4A). The Autocorrelation functions and size distribution obtained via DLS measurement are reported in Additional file 1: Fig. S1A. As it can be seen, the average hydrodynamic radius of FerOX is 5.5 ± 2.47 nm, with a highly monodisperse population of particles observed (Additional file 1: Fig. S1B). The unweighted intensity analysis shows the presence of a small population of aggregated particles with a radius of approximately 55 nm (Additional file 1: Fig. S1C). The measured surface charge of the nanodrugs was − 17.4 ± 0.361 mV, as reported in Additional file 1: Fig. S2. These results are in line with data described for HFn by several authors [41, 42].

FerOX has proven to be the optimal candidate for clinical translation in BC therapy, thanks to its capability to enhance the anticancer effectiveness of DOX, while reducing accumulation in non-target organs, thereby mitigating cardiotoxicity [36]. Here, the potential effects on preserving T-cell immune competence of FerOX was assessed.

FerOX antitumor activity has been assessed on a panel of 12 BC Patient Derived Organoids (PDO) to demonstrate its suitability to treat human cancers in comparison to free DOX and Myocet [27, 43]. Myocet has been preferred to Doxil as reference of liposomal nanoformulation since it provide equivalent toxicities despite modified DOX biodistribution [44]. FerOX showed equivalent anticancer activity to free DOX, as demonstrated in Fig. 4B, in a viability assay conducted to determine the IC_50_ of all DOX formulations. On the other side, both FerOX and free DOX exhibited greater efficacy than Myocet in inhibiting the proliferation of BC-PDO, as shown by the nearly 15- to 20-fold increase in Myocet IC_50_ (Fig. 2B). Moreover, FerOX IC_50_ values displayed an inverse correlation with TfR1 expression, confirming the capability of FerOX to mediate a specific tumor-targeted delivery of DOX, as previously described in literature (Fig. 4C) [19, 23, 24, 36, 40].

### FerOX reduces DOX internalization in peripheral blood mononuclear cells

**Fig. 5:**
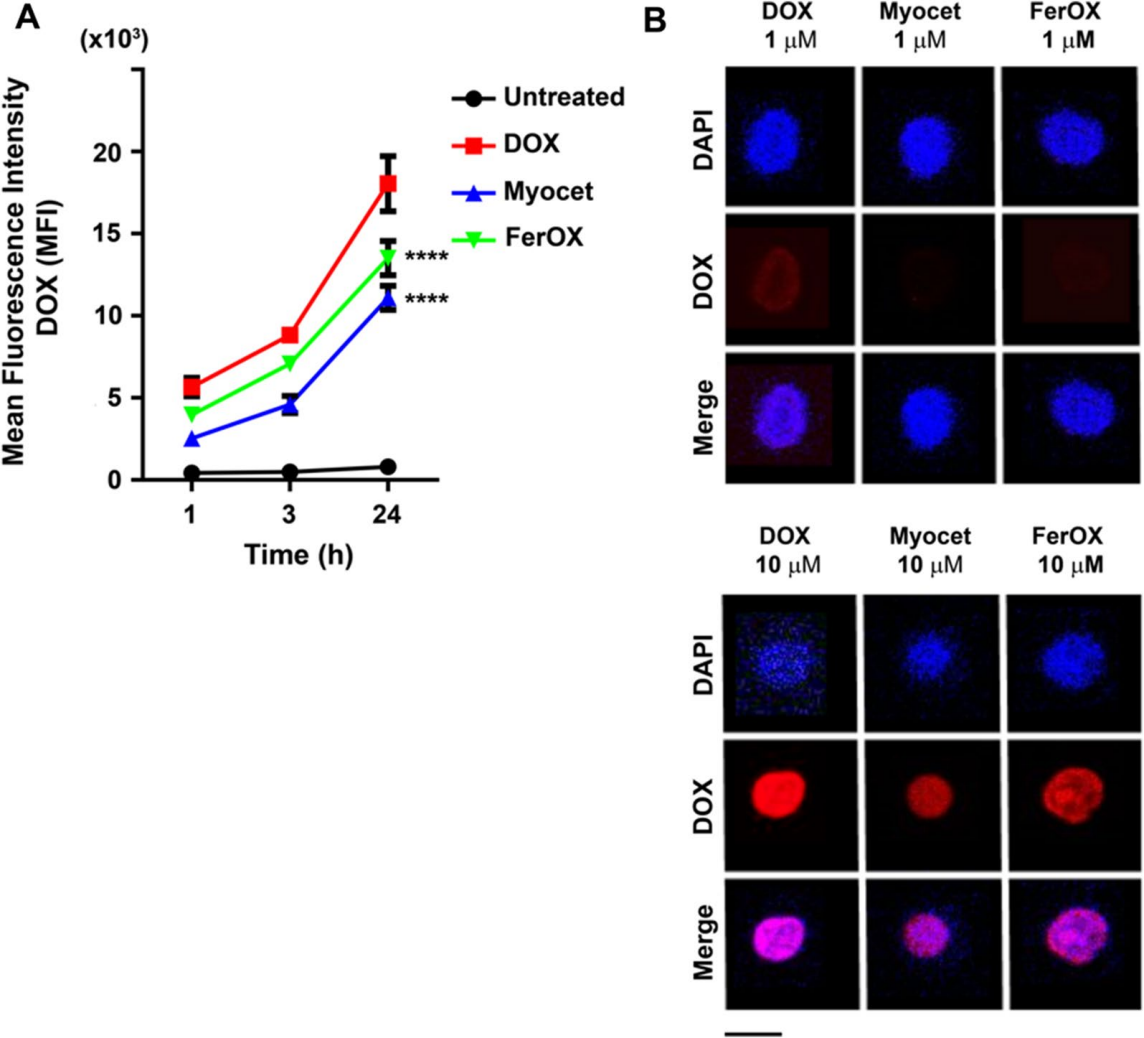
FerOX internalization in peripheral blood lymphocytes. **A** PBMC collected from healthy donors and treated with DOX, FerOX and Myocet (1 μM) for 1, 3 and 24 h to assess drug uptake. Drug uptake has been monitored during time by flow cytometry measuring DOX mean fluorescence intensity in DOX^+^ cells. Statistical significance *vs* DOX ****p < 0.001 (Two-way ANOVA). **B** PBMC treated with DOX, FerOX and Myocet at 1 and 10 μM for 24 h and acquired by confocal microscopy. Nuclei were stained with DAPI (blue), while the DOX fluorescence signal was reported in red. Scale bar = 10 µm

We then examined the influence of various nanoformulations on DOX uptake in PBMCs obtained from healthy donors to determine their potential effects on preserving T cell proliferation competence. PBMCs incubated up to 24 h with 1 μM of free DOX, FerOX or Myocet (DOX equivalents) displayed a time dependent internalization profile, as shown by MFI values of treated cells analyzed by flow-cytometry (Fig. 5A). DOX-treated PBMC displayed the higher DOX uptake in comparison to those treated with the same amount of DOX nanoformulated in FerOX (0.8-fold less) or in Myocet (0.6-fold less) (Fig. 5A). Moreover, upon internalization, DOX fluorescence was visible in the nucleus of Myocet and FerOX-treated PBMC only at the highest drug concentration of 10 μM, while in those cells treated with free DOX it is detectable even at the lowest concentration of DOX (i.e.,1 μM; Fig. 5B, C). These data evidence that free DOX has potential higher toxicity for immune cells compared with DOX nanoformulations, which significantly affect the mechanisms and kinetics of DOX uptake. Our confocal microscopy images showing free DOX uptake in PBMC (Fig. 5B) are consistent with those already published in literature across different cell types, indicating a nonspecific uptake mechanism [23]. When DOX is nanoformulated, drug uptake is reduced in cells with low TfR1 expression (i.e., PBMC), as expected in case of TfR1-mediated internalization. As reported by Human Protein Atlas, Tfr1 in CD4^+^ and CD8^+^ cells is low in comparison to monocytes and B-cells populations (Additional file 1: Fig. S3) [45]. To date, it is well reported in literature an upregulation of TfR1 expression upon CD4^+^ and CD8^+^ T cells activation consequent to mitogen treatment [46, 47]. Since the assessment of DOX-mediated proliferative impairment in PBMCs requires ConA mitogen stimulation, it would be crucial to characterize FerOX impact in proliferating PBMCs.

### FerOX displays reduced uptake in T cells, sparing central memory, effector memory and naive subpopulations.

**Fig. 6:**
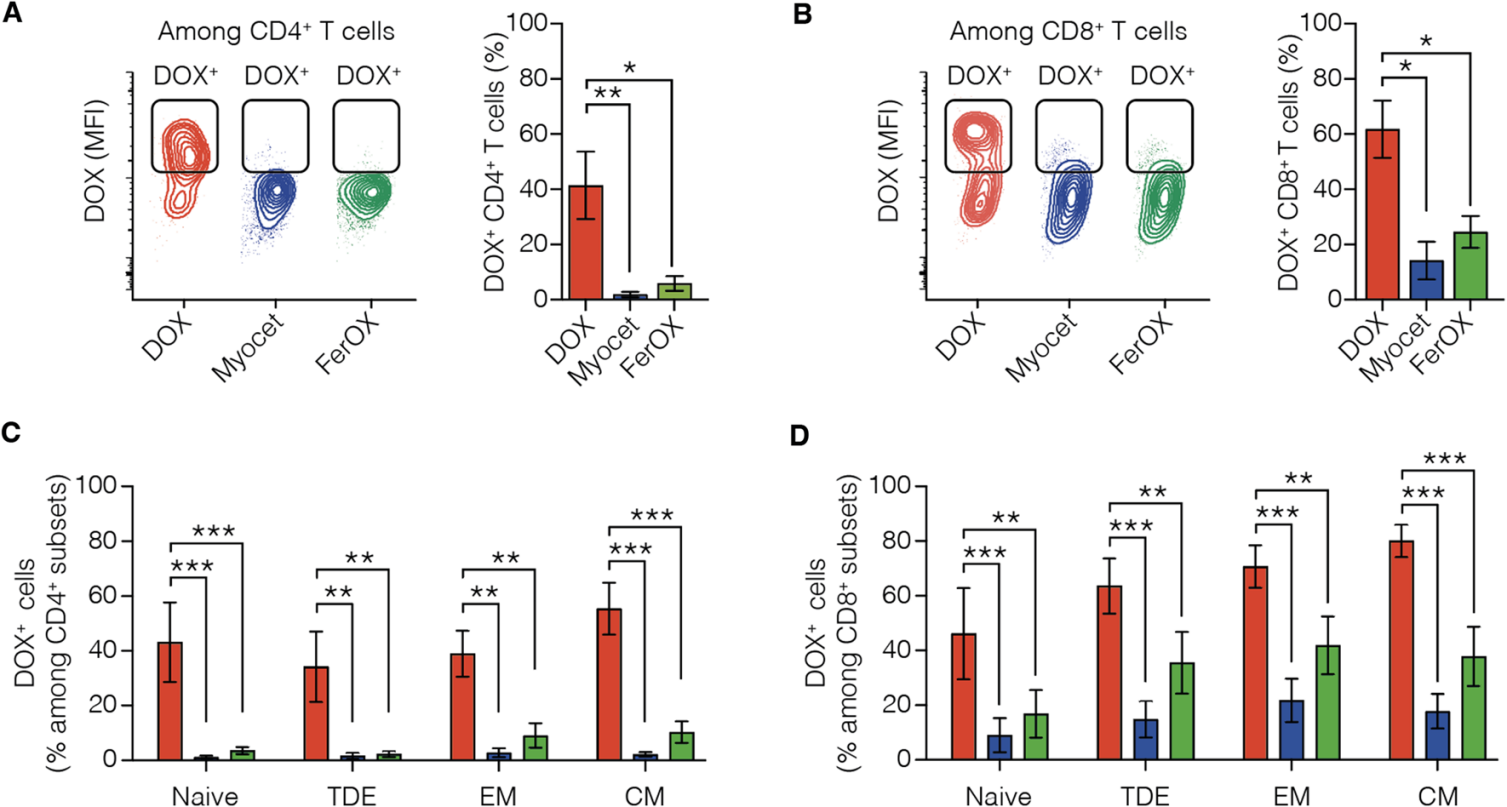
Comparison of DOX, Myocet and FerOX in vitro uptake of human peripheral T cells populations. **A** Representative plots (left panel) and frequencies (right panel) of DOX^+^ CD4^+^ T cells analyzed in human PBMCs after 24 h incubation with 5 µM of DOX, Myocet and FerOX. **B** Representative plots (left panel) and frequencies (right panel) of DOX^+^ CD8^+^ T cells analyzed in human PBMCs after 24 h incubation with 5 µM of DOX, Myocet and FerOX. Statistical significance *vs* DOX: *p < 0.05; **p < 0.01; ***p < 0.001 (One-way ANOVA). **C** Frequencies of DOX^+^ cells among Central Memory (CM), Naïve (CD197^+^ CD45RA^+^), Terminally Differentiated Effector (TDE, CD197^−^ CD45RA^+^) and Effector Memory (EM, CD197^−^ CD45RA^−^) CD4^+^ T cells. **D** Frequencies of DOX^+^ cells among CM, Naïve, TDE and EM CD8^+^ T cells. Statistical significance *vs* DOX: **p < 0.01; ***p < 0.001 (Two-way ANOVA). *n* = 4 / group, data are represented as mean ± SEM

To investigate whether a specific cell subtype exhibits a preferential uptake, the internalization of DOX was evaluated in various human T cell subpopulations following in vitro incubation with free-DOX, Myocet, or FerOX nanoformulations. We found that a population characterized by higher DOX uptake (DOX^+^) could be detected in total CD4^+^ (Fig. 6A) and in CD8^+^ T cells (Fig. 6B) when exposed to free-DOX for 24 h in vitro. In contrast, when human CD4^+^ T cells were exposed to Myocet or FerOX under the same conditions, we observed a significant 95% and 85% reduction in DOX^+^ cells, respectively (Fig. 6A). Similarly, CD8^+^ T cells exhibited a reduction of 77% and 60% in DOX^+^ cells when exposed to Myocet or FerOX, respectively (Fig. 6B). Importantly, when looking at the differential DOX uptake across different T cells subpopulations, we found that both nanoformulations significantly limited DOX uptake in CD4^+^ and CD8^+^ CM, EM, TDE and Naïve subpopulations (Fig. 6C, D). Overall, these findings may hold promise for preserving the competence of T cells to effectively mount an antitumor response.

### FerOX preserves PBMC proliferative potential

**Fig. 7:**
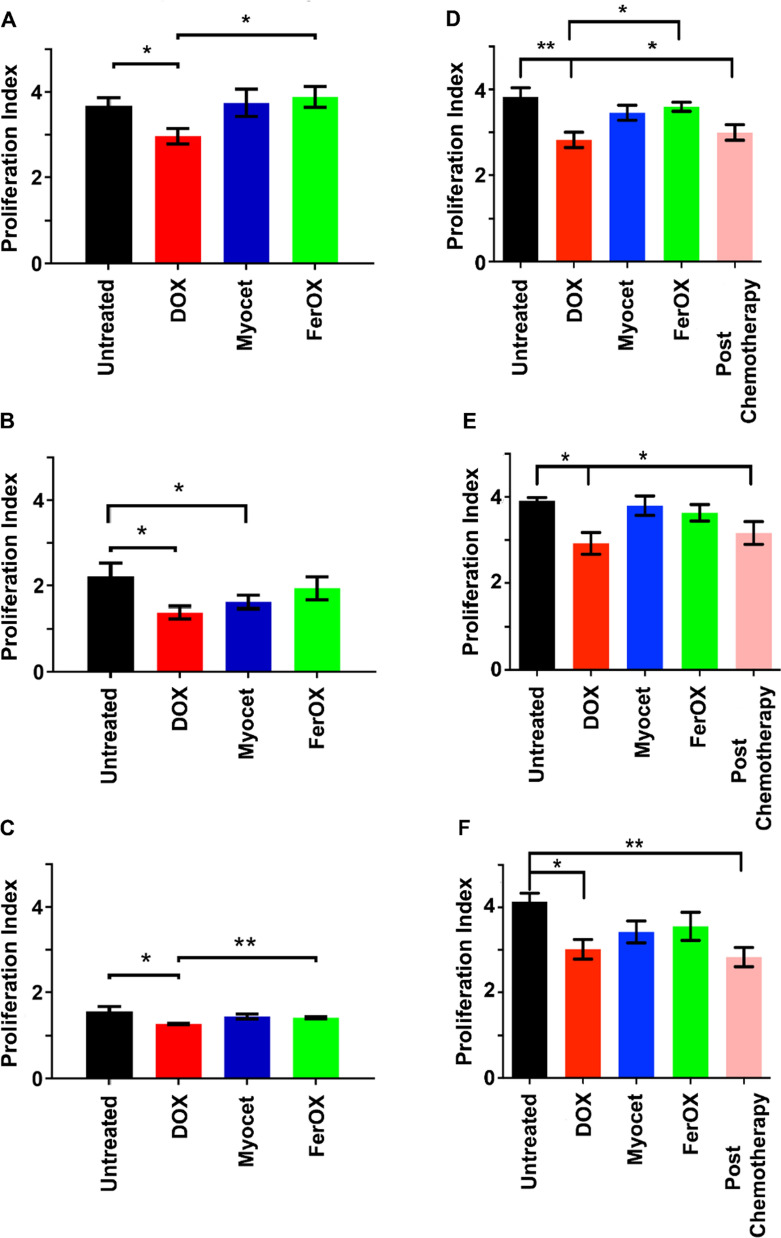
FerOX uptake preserves PBMC proliferation in vitro and ex vivo. **A** Proliferation index of PBMC collected from healthy donors, labelled with CFSE 1 μM and stimulated with ConA (5 μg/mL) after 3 h in vitro treatment with DOX, Myocet or FerOX 5 μM to simulate what occurs during the infusion time of DOX NAC. Proliferation index has been calculated using the software FlowJo (version 10). Statistical significance: *p < 0.05 (paired t-test) (n = 3). **B** Proliferation index of CD4^+^ T cells collected from healthy donors, labelled with CFSE 1 μM and stimulated with ConA (5 μg/mL) after 3 h in vitro treatment with DOX, Myocet or FerOX 5 μM calculated using the software FlowJo (version 10). Statistical significance: *p < 0.05 (paired t-test) (n = 3). **C** Proliferation index of CD8^+^ T cells collected from healthy donors, labelled with CFSE 1 μM and stimulated with ConA (5 μg/mL) after 3 h in vitro treatment with DOX, Myocet or FerOX 5 μM calculated using the software FlowJo (version 10). Statistical significance: *p < 0.05; **p < 0.01 (paired t-test) (n = 3). **D** Proliferation index of PBMC collected from BC patients before and after DOX NAC, labelled with CFSE 1 μM and stimulated with ConA (5 μg/mL). PBMC collected from BC patients before DOX NAC have been treated in vitro for 3 h with DOX, Myocet or FerOX 5 μM, in order to simulate chemotherapy. Proliferation indexes have been calculated using the software FlowJo (version 10). Statistical significance: *p < 0.05; **p < 0.01 (paired t-test) (n = 3). **E** Proliferation index of CD4^+^ T cells collected from BC patients before and after DOX NAC, labelled with CFSE 1 μM and stimulated with ConA (5 μg/mL). PBMC collected from BC patients before DOX NAC have been treated in vitro for 3 h with DOX, Myocet or FerOX 5 μM, in order to simulate chemotherapy. Proliferation indexes have been calculated using the software FlowJo (version 10). Statistical significance: *p < 0.05 (paired t-test) (n = 3). **F** Proliferation index of CD8^+^ T cells collected from BC patients before and after DOX NAC, labelled with CFSE 1 μM and stimulated with ConA (5 μg/mL). PBMC collected from BC patients before DOX NAC have been treated in vitro for 3 h with DOX, Myocet or FerOX 5 μM, in order to simulate chemotherapy. Proliferation indexes have been calculated using the software FlowJo (version 10). Statistical significance: *p < 0.05; **p < 0.01 (paired t-test) (n = 3)

In order to determine whether the decreased uptake of nanoformulated DOX in PBMCs can have a beneficial impact on T cell mediated immune response, we conducted in vitro experiments where PBMCs from healthy donors were incubated with DOX, Myocet, or FerOX. These experimental conditions were designed to simulate NAC, with a DOX concentration of 5 µM and an exposure time of approximately 3 h. In contrast to untreated cells, PBMCs treated with DOX showed a significant reduction in proliferation index (Fig. 7A). No statistically significant difference in proliferation index was found in PBMCs treated with FerOX and Myocet. Notably, exposure of PBMCs to free DOX resulted in enhanced cell death, whereas DOX nanoformulations maintained cell vitality to a degree comparable to the PBS control group (Additional file 1: Fig. S4A–C).

These findings collectively suggest that the reduced drug uptake achieved through nanoformulation not only preserves the proliferative capacity of immune cells but also maintains cell vitality during cell division. Additionally, data obtained with FerOX indirectly indicated that, even after ConA mitogen stimulation, TfR1-mediated uptake remains unaltered.

Analyzing the proliferation of T cells populations upon treatment with free or nanoformulated DOX, we confirmed the toxic activity of free DOX against both CD4^+^ and CD8^+^ cells (Fig. 7B, C). We observed that Myocet-treated cells also exhibited a decrease in proliferative potential in CD4^+^ T cells, whereas the proliferation capability of CD4^+^ T cells was unaffected in those treated with FerOX (Fig. 7B). In contrast, when considering only the CD8^+^ population, the response of Myocet and FerOX-treated cells was consistent with that observed in the entire PBMC population (Fig. 7C). To date, literature already reported that CD4^+^ T cells recover more slowly than CD8^+^ after chemotherapy, also affecting B cells activation and maturation [30].

Both FerOX and Myocet treatments were found to be safe, with a statistically significant difference in the proliferation index observed between DOX and FerOX-treated cells (Fig. 7C). Although these results suggest that DOX nanoformulations, particularly FerOX, are superior in preserving T cell immune competence and facilitating a better antitumor immune response, it is important to note that these findings were obtained from healthy donors and may not accurately reflect the behavior of PBMC in cancer patients. Therefore, we repeated similar experiments with PBMC collected from BC patients undergoing DOX NAC. We compared the proliferation index of PBMC obtained from BC patients immediately after the completion of the first cycle of DOX NAC with those obtained before DOX NAC. We also treated BC patients-derived PBMC ex vivo with DOX, Myocet, or FerOX and analyzed their effect on the proliferation index of PBMCs collected from the same patients. As expected, PBMC collected from BC patients immediately after the end of the first cycle of DOX NAC displayed an impairment of proliferative potential similar to that observed in PBMC collected from BC patients before the start of the first cycle of DOX NAC and treated ex vivo with DOX (Fig. 7D). The same behavior was observed on CD4^+^ and CD8^+^ populations (Fig. 7E, F). Importantly, PBMC treated ex vivo with Myocet and FerOX demonstrated similar proliferation activity to untreated PBMC. These results reinforce the safety of these nanoformulations and their minimal negative effect on the adaptive antitumor immune response (Fig. 7D–F). These results, coupled with the FerOX anticancer activity observed in BC-PDO, strongly highlights the promising potential of this nanoformulation and supports the need of conducting additional research to establish the suitability of FerOX for clinical applications.

## Conclusions

Besides its direct cytotoxicity against highly proliferating cancers like BC, DOX has been recently demonstrated to also exert an immunostimulatory effect in multiple ways. However, the underlying mechanism of DOX on the sensitization of BC and the effect on the adaptative immune response has not yet been elucidated and deserves to be further investigated to decipher the cellular and molecular determinants leading to complete immune-mediated tumor eradication.

This study, aimed at characterizing the interaction of DOX with primary human T cells in terms of uptake and proliferative potential, showed a global proliferative impairment in PBMCs both from healthy donors and DOX-treated BC patients. Of note, results confirmed that the higher negative impact of DOX is observed in the subset of CD8^+^ cells, as already described in literature [34, 35]. As the preservation of proliferative impact is crucial for the generation of an adaptive antitumor response in patients with BC, we evaluated the capability of alternative DOX formulations, i.e. FerOX and Myocet, in preserving T-cell immune competence. After confirming the capability of FerOX to mediate a specific tumor-targeted delivery of DOX in a panel of BC PDO, we provided evidence about the reduced undesired uptake of DOX in PBMCs, meanwhile preserving their proliferative potential. In conclusion, FerOX was found to be particularly competitive in preserving T cells allowing a potential adaptative immune response in comparison with the free drug. Overall, this study provides novel understanding on the interaction between HFn-based nanotherapeutic and the immune system and supports their potential for the development of novel nanoformulations for the immunomodulation of the tumor immune infiltrate and their significance for clinical translation in BC and other solid tumors treatments.

### Supplementary Information

**Additional file 1: Figure S1.** Dynamic Light Scattering (DLS) analysis of FerOX. Autocorrelation function (**A**), number (**B**) and intensity (**C**) averaged size distributions obtained with the CONTIN algorithm fitting. N.I.: Normalized Intensity. **Figure S2.** Zeta Potential analysis of FerOX, showing the charge distribution (**A**) and phase plot (**B**) of the measurements. **Figure S3.** Bar chart reporting the resulting transcript expression values for the transferrin receptor (TfR1) calculated as normalized transcript per million (nTPM) described in the Human Protein Atlas dataset [1] for monocytes, PBMC and T-Cells. Reprinted from [1]. **Figure S4.** Effect of DOX nanoformulation on PBMC vitality. (**A**) Experimental setup. Peripheral blood mononuclear cells (PBMCs) were isolated from three healthy donors and cells were treated with or without 5 µM of free DOX, Myocet, or FerOX. Lymphocytes were then stimulated with Concanavalin A (ConA) and incubated for 48 h. Cells were stained with Live/Dead stain reagent and analyzed by flow cytometry. (**B**-**C**) Representative histograms (**B**) and quantification (**C**) of live PBMC in the indicated conditions. *n* = 3, **p value < 0.01, one-way Kruskal–Wallis ANOVA test with Dunnett correction for multiple comparisons. **Table S1.** Demographic and clinicopathological characteristics of patients and healthy donors.

## Data Availability

Data available after publication in a publicly accessible repository https://doi.org/10.13130/RD_UNIMI/NYZYIZ.

## Abbreviations

Ag: Antigens
BC: Breast cancer
BSA: Bovine serum albumin
CFSE: Carboxyfluorescein succinimidyl ester
CM: Central memory
ConA: Concanavalin A
DOX: Doxorubicin
EE: Encapsulation efficiency
EF: Effector memory
FerOX: Doxorubicin formulation in ferritin nanocages
HER2: Human epidermal growth factor receptor 2
HFn: Human ferritin
ICCD: Immunogenic cancer cell death
LC: Loading capacity
MFI: Mean fluorescence intensity
NAC: Neoadjuvant chemotherapy
NP: Nanoparticles
PBMC: Patient-derived peripheral blood mononuclear cells
PBS: Phosphate-buffered saline
PDO: Patient-derived organoids
PFA: Paraformaldehyde
RT: Room temperature
SEM: Standard error of mean
TDE: Terminally differentiated effector
TfR1: Transferrin receptor 1
TNBC: Triple negative breast cancer
