# Publisher Correction: Impact of doxorubicin-loaded ferritin nanocages (FerOX) vs. free doxorubicin on T lymphocytes: a translational clinical study on breast cancer patients undergoing neoadjuvant chemotherapy

**DOI:** 10.1186/s12951-024-02532-2

**Published:** 2024-05-27

**Authors:** Marta Sevieri, Francesco Andreata, Francesco Mainini, Lorena Signati, Francesca Piccotti, Marta Truffi, Arianna Bonizzi, Leopoldo Sitia, Claudia Pigliacelli, Carlo Morasso, Barbara Tagliaferri, Fabio Corsi, Serena Mazzucchelli

**Affiliations:** 1 Dipartimento di Scienze Biomediche e Cliniche, Università di Milano, Milan, 20157 Italy; 2 Istituti Clinici Scientifici Maugeri IRCCS, Pavia, 27100 Italy; 3 Division of Immunology, Transplantation, and Infectious Diseases, IRCCS San Raffaele Scientific Institute, Milan, Italy; 4 Laboratory of Supramolecular and Bio-Nanomaterials (SBNLab), Department of Chemistry, Materials, and Chemical Engineering “Giulio Natta”, Politecnico di Milano, Milan, 20131 Italy

**Correction: Journal of Nanobiotechnology (2024) 22:184**

10.1186/s12951-024-02441-4

Following publication of the original article details for affiliations of all authors were incorrectly given as:

1. Department of Biomedical and Clinical Sciences, Università di Milano, 20157 Milan, Italy.

2. Division of Immunology, Transplantation, and Infectious Diseases, IRCCS San Rafaele Scientifc Institute, Milan, Italy.

3. Istituti Clinici, Scientifci Maugeri IRCCS, 27100 Pavia, Italy.

4. Laboratory of Supramolecular and Bio-Nanomaterials (SBNLab), Department of Chemistry, Materials, and Chemical Engineering “Giulio Natta”, Politecnico di Milano, 20131 Milan, Italy.

But should have been:

1. Dipartimento di Scienze Biomediche e Cliniche, Università di Milano, Milan 20157, Italy.

2. Istituti Clinici Scientifici Maugeri IRCCS, Pavia 27100, Italy.

3. Division of Immunology, Transplantation, and Infectious Diseases, IRCCS San Raffaele Scientific Institute, Milan, Italy.

4. Laboratory of Supramolecular and Bio-Nanomaterials (SBNLab), Department of Chemistry, Materials, and Chemical Engineering “Giulio Natta”, Politecnico di Milano, Milano 20131, Italy.

The original article has been updated.

