# Supplementary material for: Impact of doxorubicin-loaded ferritin nanocages (FerOX) vs. free doxorubicin on T lymphocytes: a translational clinical study on breast cancer patients undergoing neoadjuvant chemotherapy
Source: J Nanobiotechnology. 2024 Apr 15;22:184. doi: 10.1186/s12951-024-02441-4 (PMC11020177; doi:10.1186/s12951-024-02441-4)
Supplement: Supplementary file 1 — Additional file 1: Figure S1. Dynamic Light Scattering (DLS) analysis of FerOX. Autocorrelation function (A), number (B) and intensity (C) averaged size distributions obtained with the CONTIN algorithm fitting. N.I.: Normalized Intensity. Figure S2. Zeta Potential analysis of FerOX, showing the charge distribution (A) and phase plot (B) of the measurements. Figure S3. Bar chart reporting the resulting transcript expression values for the transferrin receptor (TfR1) calculated as normalized transcript per million (nTPM) described in the Human Protein Atlas dataset [1] for monocytes, PBMC and T-Cells. Reprinted from [1]. Figure S4. Effect of DOX nanoformulation on PBMC vitality. (A) Experimental setup. Peripheral blood mononuclear cells (PBMCs) were isolated from three healthy donors and cells were treated with or without 5 µM of free DOX, Myocet, or FerOX. Lymphocytes were then stimulated with Concanavalin A (ConA) and incubated for 48 h. Cells were stained with Live/Dead stain reagent and analyzed by flow cytometry. (B-C) Representative histograms (B) and quantification (C) of live PBMC in the indicated conditions. n = 3, **p value < 0.01, one-way Kruskal–Wallis ANOVA test with Dunnett correction for multiple comparisons. Table S1. Demographic and clinicopathological characteristics of patients and healthy donors. [file 12951_2024_2441_MOESM1_ESM.docx]

***Impact of Doxorubicin-loaded Ferritin Nanocages (FerOX) vs. free doxorubicin on T lymphocytes: a translational clinical study on breast cancer patients undergoing neoadjuvant chemotherapy***

Marta Sevieri,^1,*^ Francesco Andreata,^3,*^ Francesco Mainini,^1^ Lorena Signati,^1,2^ Francesca Piccotti,^2^ Marta Truffi,^2^ Arianna Bonizzi,^2^ Leopoldo Sitia,^1^ Claudia Pigliacelli,^4^ Carlo Morasso,^2^ Barbara Tagliaferri,^2^ Fabio Corsi,^1,2,#^ Serena Mazzucchelli ^1,#^

1. Dipartimento di Scienze Biomediche e Cliniche, Università di Milano, Milan 20157, Italy.
2. Istituti Clinici Scientifici Maugeri IRCCS, Pavia 27100, Italy.
3. Division of Immunology, Transplantation, and Infectious Diseases, IRCCS San Raffaele Scientific Institute, Milan, Italy.
4. Laboratory of Supramolecular and Bio-Nanomaterials (SBNLab), Department of Chemistry, Materials, and Chemical Engineering “Giulio Natta”, Politecnico di Milano, Milano 20131, Italy

^#^ Correspondence to: Fabio Corsi, [fabio.corsi@unimi.it](mailto:fabio.corsi@unimi.it); Serena Mazzucchelli, [serena.mazzucchelli@unimi.it](mailto:serena.mazzucchelli@unimi.it)

*These authors contributed equally to the work.

## **Additional Material**

In this study, we considered significant works regarding the state of the art on the use of DOX and its impact on the immune system published between 2004 and now. We subsequently focused on studies published between 2014 and 2023 reporting excellent investigations of nanoparticles for enhanced antitumor therapeutic potential with reduced toxicity. Of note, most of the studies concerning DOX-loaded ferritin nanoparticles, as the one proposed by our group, have been published in the last 5 years, demonstrating the strong attractiveness of this topic.

| 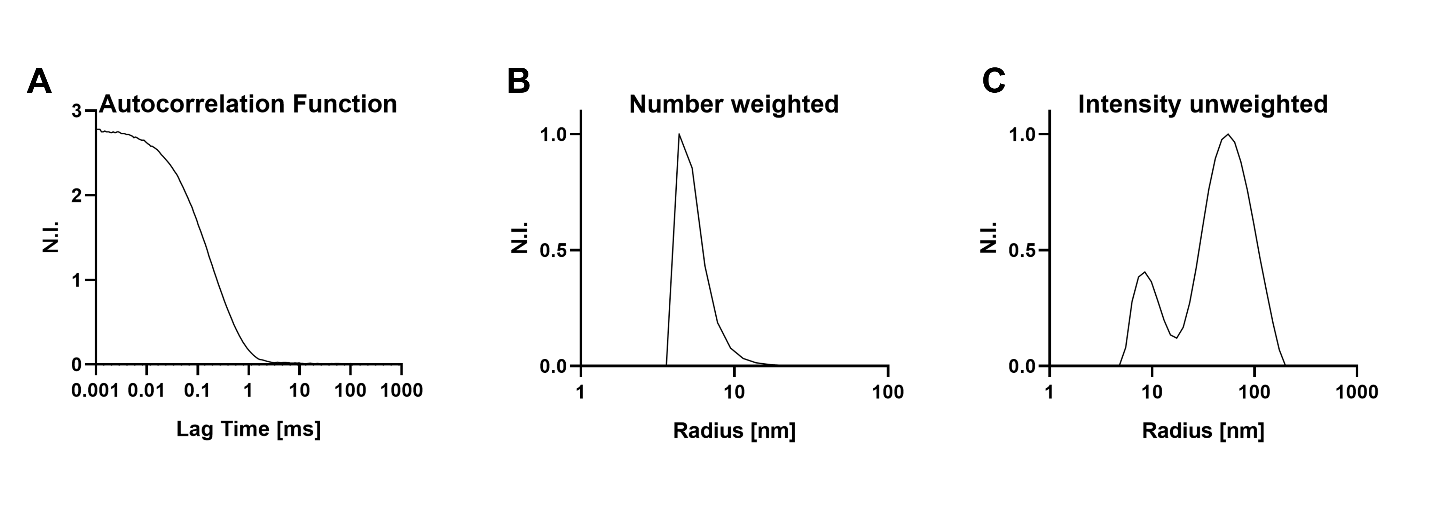 | |  |
| --- | --- | --- |
| **Figure S1**. Dynamic Light Scattering (DLS) analysis of FerOX . Autocorrelation function (**A**), number (**B**) and intensity (**C**) averaged size distributions obtained with the CONTIN algorithm fitting. N.I.: Normalized Intensity. | |  |
| **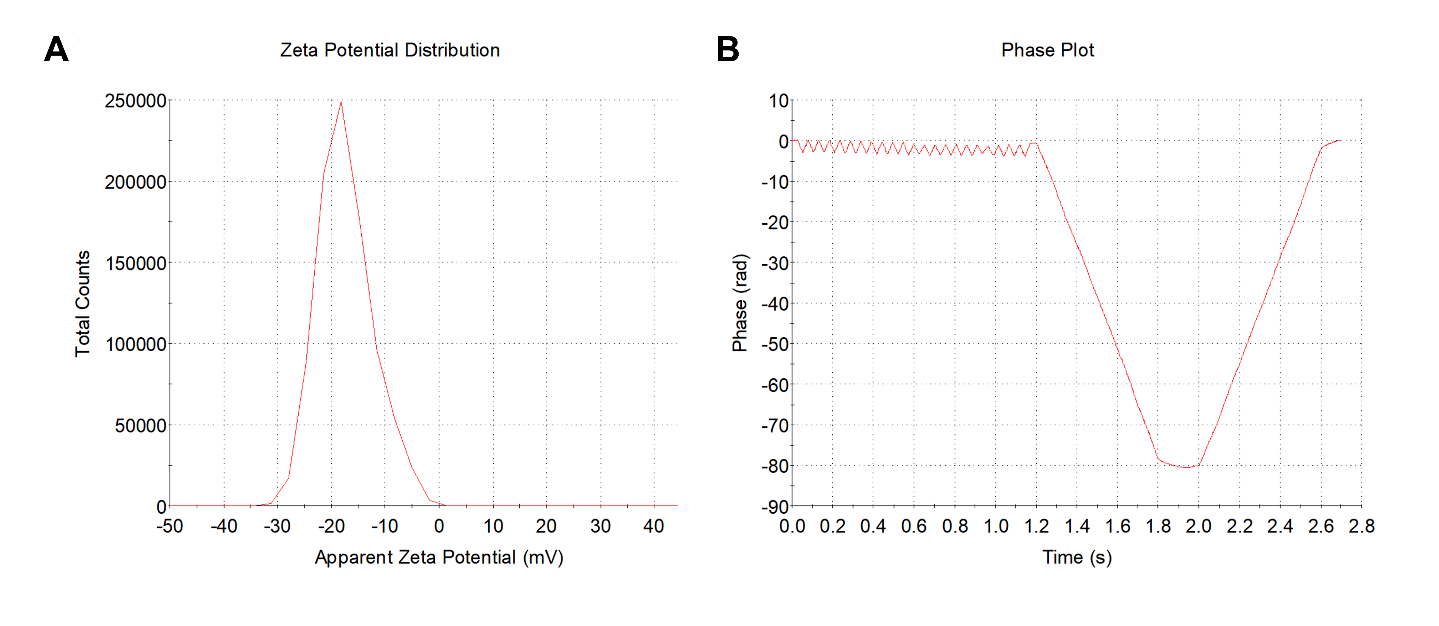**  **Figure S2.** Zeta Potential analysis of FerOX , showing the charge distribution (**A**) and phase plot (**B**) of the measurements. | |  |
| **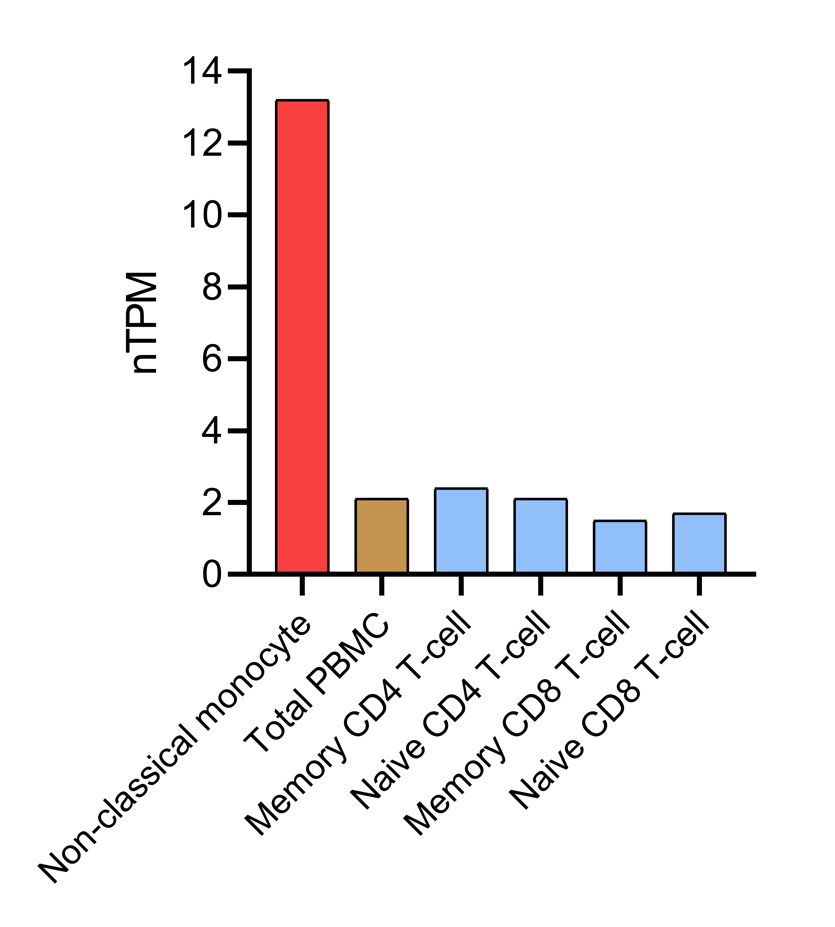** | | |
|  | **Figure S3**. Bar chart reporting the resulting transcript expression values for the transferrin receptor (TfR1) calculated as normalized transcript per million (nTPM) described in the Human Protein Atlas dataset [1] for monocytes, PBMC and T-Cells. Reprinted from [1]. | |

| 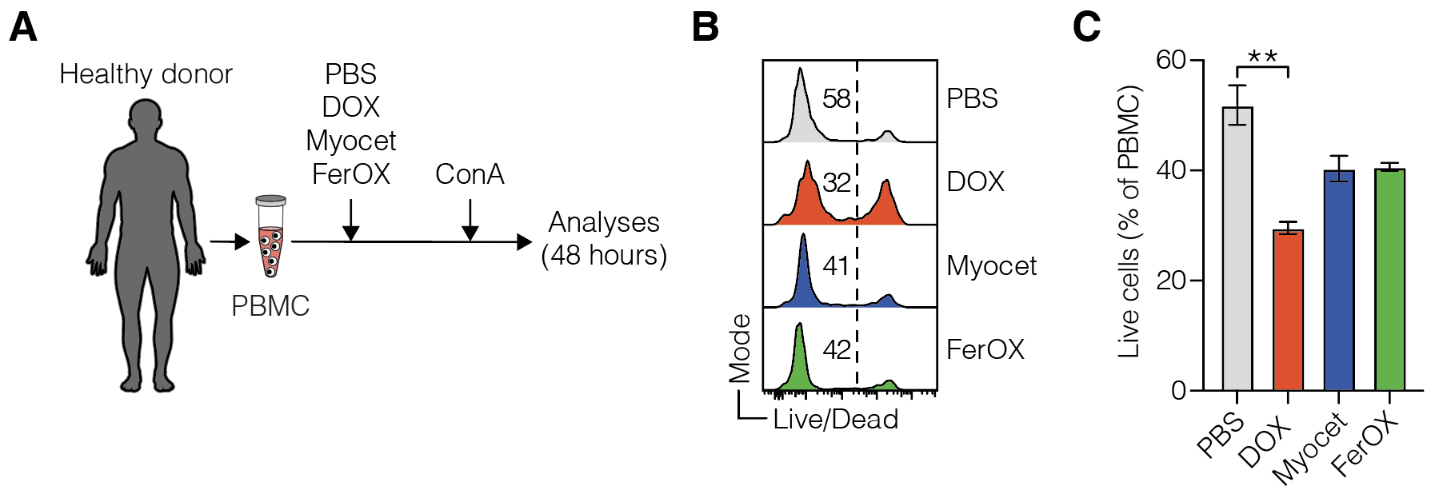 |
| --- |
| **Figure S4**. **Effect of DOX nanoformulation on PBMC vitality.**  (**A**) Experimental setup. Peripheral blood mononuclear cells (PBMCs) were isolated from three healthy donors and cells were treated with or without 5µM of free DOX, Myocet, or FerOX. Lymphocytes were then stimulated with Concanavalin A (ConA) and incubated for 48 hours. Cells were stained with Live/Dead stain reagent and analyzed by flow cytometry. (**B**-**C**) Representative histograms (**B**) and quantification (**C**) of live PBMC in the indicated conditions. *n* = 3, **p value < 0.01, one-way Kruskal-Wallis ANOVA test with Dunnett correction for multiple comparisons. |

**Table S1.** Demographic and clinicopathological characteristics of patients and healthy donors.

| **Donor Characteristics** | **BC Patients** **n = 30** | **Healthy Donors** **n = 6** |
| --- | --- | --- |
| **Age** Median ± st.dev. Range | 55,07 ± 9,23 27-72 | 31,17 ± 5,12 24-39 |
| **Sex** | F = 30 | F = 3 M = 3 |
| **Histological Type** | IDC = 28 ILC = 2 |  |
| **Molecular Subtype** | Luminal A = 2 Luminal B HER2- = 6  Luminal B HER2+ = 9 HER2-enriched = 6 TNBC = 7 |  |
| **Histological Grading** | G1 = 1 G2 = 13 G3 = 16 |  |
| **NAC Type** | AC = 1 AC + Taxane = 11 AC + Taxane + TZ = 16 AC + Taxane + Carboplatin = 2 |  |
| **NAC Response** | pCR = 14 non pCR =16 |  |

*Abbreviations:*

BC, breast cancer; IDC, invasive ductal carcinoma; ILC, invasive lobular carcinoma; TNBC, triple negative breast cancer; NAC, neoadjuvant chemotherapy; AC, anthracycline; TZ, Trastuzumab; pCR, pathological complete response.

*References:*

1. Human Protein Atlas proteinatlas.org [Internet]. Available at: https://www.proteinatlas.org/ENSG00000072274-TFRC/immune+cell
